# Core outcome sets and trial registries

**DOI:** 10.1186/s13063-015-0738-6

**Published:** 2015-05-14

**Authors:** Mike Clarke, Paula Williamson

**Affiliations:** Northern Ireland Network for Trials Methodology Research, Centre for Public Health, Institute of Clinical Sciences, Block B, Queens University Belfast, Royal Hospitals, Grosvenor Road, Belfast, BT12 6BJ UK; Department of Biostatistics, University of Liverpool, 1st floor Duncan Building, Daulby Street, Liverpool, L69 3GA UK

## Abstract

Some reasons for registering trials might be considered as self-serving, such as satisfying the requirements of a journal in which the researchers wish to publish their eventual findings or publicising the trial to boost recruitment. Registry entries also help others, including systematic reviewers, to know about ongoing or unpublished studies and contribute to reducing research waste by making it clear what studies are ongoing. Other sources of research waste include inconsistency in outcome measurement across trials in the same area, missing data on important outcomes from some trials, and selective reporting of outcomes. One way to reduce this waste is through the use of core outcome sets: standardised sets of outcomes for research in specific areas of health and social care. These do not restrict the outcomes that will be measured, but provide the minimum to include if a trial is to be of the most use to potential users. We propose that trial registries, such as ISRCTN, encourage researchers to note their use of a core outcome set in their entry. This will help people searching for trials and those worried about selective reporting in closed trials. Trial registries can facilitate these efforts to make new trials as useful as possible and reduce waste. The outcomes section in the entry could prompt the researcher to consider using a core outcome set and facilitate the specification of that core outcome set and its component outcomes through linking to the original core outcome set. In doing this, registries will contribute to the global effort to ensure that trials answer important uncertainties, can be brought together in systematic reviews, and better serve their ultimate aim of improving health and well-being through improving health and social care.

## Background

There are many reasons for registering trials. Some of these might be considered as self-serving, such as satisfying the requirement that trials be prospectively registered before the first patient is randomised if the researchers wish to publish their findings in certain journals [1] or publicising the trial to try to boost recruitment. Registry entries also help others to know what studies are ongoing and will help future systematic reviewers to examine the risks to their review of reporting biases and to see what was planned at the start of a trial. Trial registration contributes to reducing waste in research by helping to avoid unnecessary duplication of effort [2] and making it easier for health and social care research to be cumulative [3]. It is also an opportunity to focus the attention of the researchers on ensuring that their trial is designed in a way that will maximise its potential for impact on future decisions and policy. To maximise impact, however, it is vital that the outcomes that will be measured and reported are carefully chosen and will be relevant to those making decisions and setting policy in the future. This commentary highlights how careful consideration of core outcome sets when registering trials might help with choosing outcomes and how a trial registry such as ISRCTN might assist with this.

Some of the advantages of trial registration rely on clarity in the registry entry, so that the trial design is clear; furthermore, preparing the entry may be an opportunity for the researchers to give careful consideration to each of its elements. Of course, much of this consideration is likely to have taken place during earlier stages in the trial such as during the preparation of the trial protocol, when making submissions for funding, while seeking ethical approval and when satisfying other requirements such as research governance. The researchers should have already considered the scientific, ethical and environmental justification for the trial. Ideally, this might have been through a systematic review of the existing evidence to elucidate the need for the trial and to demonstrate the uncertainty that it seeks to resolve [4]. In many areas of health and social care, one of the things that a systematic review is likely to have made even clearer to the researchers is the problem caused by inconsistencies in how earlier research has chosen, measured and reported outcomes.

## Main text

### The need to standardise outcomes across research in the same area

Heterogeneity in how trials have been done can bring richness to systematic reviews and meta-analyses by testing different forms of an intervention, action or strategy and by testing these in different settings and with different populations. However, heterogeneity in the outcomes can hamper or prevent the ability of reviewers to pool the findings of studies in meta-analyses [5, 6] and will impede efforts to streamline the systematic reviewing process [7]. Inconsistency in outcome measurement across trials in the same area, a lack of data on important outcomes from all trials, and selective reporting of outcomes compound the problems faced by users of research [8, 9]. One way to reduce this waste is through the development of agreed upon, standardised sets of outcomes for research in specific areas of health and social care. The implementation of these core outcome sets in all trials in a particular topic area would maximise the ability of the researchers themselves, reviewers and others to compare, contrast and combine the findings of the separate trials. Their use is now encouraged by funders of research, including the National Institute for Health Research in the UK [10].

### Core outcome sets

If core outcome sets are to meet their potential to help researchers ensure that the outcomes they measure and report are the most relevant to users of their trial, these outcome sets need to be developed in a collaborative way with appropriate representation from key stakeholders. This is likely to include practitioners, patients and others users of health and social care services, as well as researchers, research funders, policy makers and those who pay for health and social care services. Although sporadic examples of what might now be considered to be core outcome sets have existed for some time [11], and well-established ones, such as OMERACT for rheumatoid arthritis, appear to have had an impact on trial design [12], the use of these sets is still in a period of relative infancy. Over the last five years, the COMET Initiative has been seeking to support others in the development and uptake of core outcome sets across health and social care [13] and has shown the wide variety of methods that have been used to develop them [14]. Guidance is beginning to appear [15], and a recent systematic review identified nearly 200 examples [14].

### Using core outcome sets when registering a trial

These core outcome sets have been brought together in a unique resource in the COMET database, which is freely available on the internet (www.cometinitiative.org/studies/search). This makes it much easier for researchers to identify a core outcome set if one already exists, as is evidenced by the fact that more than 25,000 records had to be checked as part of the systematic review [14]. As part of the planning for their trial, researchers who wish to maximise the potential impact of their findings need to ensure that they will measure outcomes that will make it easier to show the relevance of those findings. They can do this by determining whether or not a core outcome exists, and in some cases, people embarking on a programme of research have developed a core outcome set to ensure the relevance of the outcomes they will measure and report [16]. Researchers should consider a core outcome set when designing their trial. This does not restrict the outcomes that they will measure but does provide them with the minimum outcomes that they need to include. They remain able to include additional outcomes, especially those that may be particularly pertinent to their study.

Then, when it comes to registering a trial, the trial registry should encourage researchers to note their use of the core outcome set and to specify each of the outcomes from the core set, as well as any additional outcomes, that they will measure. Specifying the outcomes from the core outcome set and using the terms for these outcomes that were used in that core outcome set will facilitate searching by users of the registry. In fact, this could be made even easier if the registry entry could be automatically populated with the outcomes through a dynamic link to the core outcome set cited by the researchers. Another benefit from considering the core outcome set in the design and registration of their trial is that researchers may be helped in their choice of primary outcomes. These outcomes are likely to be used to determine the sample size for the trial but also will highlight the areas on which the researchers will focus. For example, the primary outcomes will be those on which they will focus most when seeking to minimise loss to follow-up and missing data.

When others inspect the registry entry for a closed trial, the explicit use of a core outcome set will facilitate one of the other benefits of a prospective registry: the ability to determine the risk of bias from measured, but not reported, outcomes. If outcomes from the core outcome set are not subsequently included in reports of the trial, this will help reviewers and others to investigate the potential impact of selective reporting bias on any decisions they wish to make.

## Conclusion

Trial registries can facilitate these efforts to make new trials as useful as possible and to reduce waste. Guidelines for reporting protocols for research acknowledge the potential value of core outcome sets [17], and an important next step would be for trial registries to do likewise. The section in the registry entry for the outcomes for the trial could prompt the researcher to consider using a core outcome set, facilitate the specification of that core outcome set and its component outcomes through linking to the original core outcome set, and encourage the choice of one or more primary outcomes from within the core outcome set. In doing this, trial registries will contribute to the global effort to ensure that trials answer important uncertainties and can be brought together in systematic reviews. We welcome comments on these suggestions and on how greater use of core outcome sets in trial registries might help researchers to achieve their ultimate aim of improving health and well-being through improving health and social care.

## Abbreviations

COMET: Core Outcome Measures in Effectiveness Trials
ISRCTN: International Standard Randomised Controlled Trial Number
OMERACT: Outcome Measures in Rheumatology
